# A dual fluorescent multiprobe assay for prion protein genotyping in sheep

**DOI:** 10.1186/1471-2334-5-13

**Published:** 2005-03-15

**Authors:** Mario Van Poucke, Jo Vandesompele, Marc Mattheeuws, Alex Van Zeveren, Luc J Peelman

**Affiliations:** 1 Department of Animal Genetics and Breeding, Faculty of Veterinary Medicine, Ghent University, Heidestraat 19, B-9820 Merelbeke, Belgium; 2 Center for Medical Genetics, Faculty of Medicine and Health Sciences, Ghent University Hospital, De Pintelaan 185, B-9000 Ghent, Belgium

## Abstract

**Background:**

Scrapie and BSE belong to a group of fatal, transmissible, neurodegenerative diseases called TSE. In order to minimize the risk of natural scrapie and presumed natural BSE in sheep, breeding programmes towards TSE resistance are conducted in many countries based on resistance rendering *PRNP *polymorphisms at codons 136 (A/V), 154 (R/H) and 171 (R/H/Q). Therefore, a reliable, fast and cost-effective method for routine *PRNP *genotyping in sheep, applicable in standard equipped molecular genetic laboratories, will be a vital instrument to fulfill the need of genotyping hundreds or thousands of sheep.

**Methods:**

A dual fluorescent multiprobe assay consisting of 2 closed tube PCR reactions containing respectively 4 and 3 dual-labelled fluorescent ASO probes for the detection in real-time of the 7 allelic variants of sheep *PRNP *mentioned above.

**Results:**

The assay is succesfully performed using unpurified DNA as a template for PCR, without any post-PCR manipulations and with semi-automatic determination of the *PRNP *genotypes. The performance of the assay was confirmed via PCR-RFLP and sequencing in a cross-validation study with 50 sheep.

**Conclusions:**

We report the development and validation of a robust, reliable and reproducible method for *PRNP *genotyping of a few to many sheep samples in a fast, simple and cost-effective way, applicable in standard equipped molecular genetic laboratories. The described primer/probe design strategy can also be applied for the detection of other polymorphisms or disease causing mutations.

## Background

TSE or prion diseases are a group of fatal, transmissible, neurodegenerative diseases occurring in human (e.g. CJD and vCJD) and animals (e.g. scrapie in sheep and BSE in cattle). These complex diseases (for reviews see [1]) are characterized by an accumulation in the central nervous system of PrP^Sc^, a disease-causing isoform of the host encoded cellular prion protein, termed PrP^C ^[2]. In sheep, it is known that polymorphisms at codons 136 (A/V), 154 (R/H) and 171 (R/H/Q) of *PRNP*, the gene encoding PrP^C^, are associated with TSE resistance/susceptibility [3].

Based on epidemiological evidences, it is almost certain that scrapie is not transmissible to human, in contrast to BSE, where there is strong evidence that it causes vCJD in human [4]. Moreover, also sheep can be infected by BSE via experimental transmission, although the ARR/ARR *PRNP *genotype seems to render the highest degree of resistance to both classical scrapie and BSE [5]. Because there is great concern about the possibility that sheep are also infected by BSE in nature, the EU forces all EU countries to conduct breeding programmes in order to select for sheep with the most TSE resistant genotypes to minimize the risk of TSE, or eventually eradicate the disease from sheep stocks [6].

In order to accomplish this goal, a reliable, fast and cost-effective method for *PRNP *genotyping in sheep is indispensable. To date, a number of methods are used, including sequencing [7], PCR-RFLP [8], DGGE analysis [9], primer extension assay [10], ARMS [11], TaqMan-MGB assay [12] and reverse hybridization (Stefan Roels, personal communication). Here we report the development and validation of a new, dual fluorescent multiprobe assay, consisting of 2 closed tube PCR reactions containing respectively 4 and 3 dual-labelled fluorescent ASO probes for the detection in real-time of the 7 allelic variants of sheep *PRNP *mentioned above.

It is important to note that the last few years there are more and more reports of sheep that are infected with atypical scrapie, including sheep with the ARR/ARR genotype. New insights in this new form of scrapie, that seems to be associated with *PRNP *codons 141 and 154 [13], will probably have a major impact on the ongoing breeding programmes for reduced TSE susceptibility and will probably result in the development of adapted genotyping methods.

## Methods

### DNA preparation from blood samples

Whole blood samples from 58 Belgian sheep (Texel, Suffolk, Swifter, Bleu du Maine, Ardense Voskop, Vlaams Schaap, Lakens Schaap, Belgisch Melkschaap) were collected in tubes containing anti-coagulant and stored at -20°C. Two hundred μl blood was washed 3 times with 500 μl TE (10 mM Tris-HCl pH 8; 1 mM EDTA pH 8) to roughly clean-up the white blood cells. The pellet was resuspended in 100 μl Lysis Buffer K (10 mM Tris-HCl pH 8; 50 mM KCl; 0.5% Tween 20) supplemented with 100 μg/ml proteinase K (Roche Diagnostics, Belgium) and incubated for 45' at 56°C to release the DNA. The lysate was incubated for 10' at 95°C to inactivate the proteinase K and centrifugated at 16.100 × g for 1' to pellet down the cell debris. This unpurified DNA solution has an average concentration of 50 ng/μl.

### PCR primer and probe design

Primers and dual-labelled ASO probes were designed with Primer Express software version 2.0 (Applied Biosystems, USA) and synthesized by Sigma-Genosys (UK). Their sequences and T_m _(calculated via T_m _Utility [14]) are listed in Table 1.

**Table 1:** An overview of the primers and probes used in the dual fluorescent multiprobe assay

**Primer/Probe Name**	**Sequence (5' → 3')**	**Length (bp)**	**T_m _(°C)**	**_av_T_m _(°C)**
Forward primer	GCCTTGGTGGCTACATG	17	59.35	-
Reverse primer	CTGTGATGTTGACACAGTCAT	21	59.60	-
A_136_-probe	*FAM*-TGCTCATGGCACTTCCCA-*BHQ1*	18	62.98	56.27
V_136_-probe	*HEX*-CTGCTCATGACACTTCCCAG-*BHQ1*	20	61.86	55.33
R_154_-probe	*TexasRed*-CCGTTACTATCGTGAAAACATGTAC-*BHQ2*	25	61.08	56.59
H_154_-probe	*Cy5*-CCGTTACTATCATGAAAACATGTACC-*BHQ2*	26	61.06	56.67
R_171_-probe	*FAM*-CCAGTGGATCGGTATAGTAACCA-*BHQ1*	23	62.27	57.19
H_171_-probe	*HEX*-AGACCAGTGGATCATTATAGTAACCA-*BHQ1*	26	61.96	57.66
Q_171_-probe	*TexasRed*-CCAGTGGATCAGTATAGTAACCAGA-*BHQ2*	25	62.07	58.46

Fluorescent labels and quenchers are in italic, SNPs are underlined. _(av)_T_m _was calculated using T_m _Utility [14].

The primers generate a 180-bp amplicon of *PRNP *exon 3 containing codons 136, 154 and 171. The primer sequences contain no described SNPs, maximizing the range of animals that can be tested, and contain no more than 2 Gs or Cs in the last 5 bp and no more than 3 consecutive Gs, minimizing self-complementarity and the formation of primer-dimers and hairpin structures.

A dual-labelled ASO probe was designed for each of the 7 allelic variants, in which the SNP is localized approximately in the middle of the sequence. The probe sequence does not start with a G, preventing quenching of the fluorophore, and contains no more than 3 consecutive Gs. For all possible candidate probes of each SNP, the melting temperature was calculated for the perfect match (T_m_) and for the single mismatch (_av_T_m_) with the other allelic variant. Those probes were selected for which the T_m _with the perfect match was higher than the T_m _of the primers and for which the _av_T_m _with the single mismatch was lower than the T_m _of the primers (see Table 1).

In PCR 1, the 4 allelic variants of codons 136 (A/V) and 154 (R/H) are determined simultaneously with 4 allele-specific probes, each containing a different fluorophore. A combination of the fluorophores FAM/HEX/TexasRed/Cy5 was chosen as described by Ugozzoli et al. [15]. In PCR 2, the 3 allelic variants of codon 171 (R/H/Q) are determined simultaneously with 3 allele-specific probes, containing resp. FAM/HEX/TexasRed as fluorophore. The fluorophores FAM/HEX are quenched with BHQ1, and TexasRed/Cy5 with BHQ2.

### Quality test for dual-labelled ASO probes

In order to check the quality of the probes, 1 μl 10 μM probe was digested with 1 U of RQ1 DNase (Promega, USA) for 30' at 37°C in a final volume of 10 μl. After adding 40 μl of 10 mM Tris-HCl pH 8, the fluorescence was measured by using the Imaging Services of the iCycler iQ Real-Time PCR Detection System (Bio-Rad Laboratories, USA) with 50 μl of the Bio-Rad calibration dye as a reference in a separate tube.

### Dual fluorescent multiprobe assay

Both PCRs of the assay were performed in the iCycler iQ Real-Time PCR Detection System (Bio-Rad Laboratories, USA) using normal PCR tubes (ABgene, UK), in a total volume of 15 μl containing iQ Supermix (50 mM KCl, 20 mM Tris-HCl pH 8.4, 0.8 mM dNTPs, 0.375 U iTaq DNA polymerase, 3 mM MgCl_2 _and stabilizers; Bio-Rad Laboratories, USA), 400 nM of each primer and probe, and ~150 ng DNA. The real-time PCR program for both reactions consists of an iTaq DNA polymerase activation and DNA denaturation step (3' at 95°C), followed by 40 amplification cycles (denaturation for 20" at 95°C and annealing-elongation for 40" at 62°C). The fluorescent signals, generated by the cleavage of the dual-labelled ASO probes, were detected in real-time during the annealing-elongation step.

Data was analysed by the iCycler iQ Real-Time PCR Detection System Software version 3.0a (Bio-Rad Laboratories, USA). For every probe, RFU-values were measured every cycle and after background normalization plotted against the cycle number. Based on these real-time amplification plots, Ct-values were calculated in the PCR Quantification tab of the Data Analysis module by user defined assignation of the Baseline Cycles and the Threshold position. The obtained data, Ct-values for detection and 'N/A' for non-detection, was then copied to a Microsoft Excel-spreadsheet [see Additional file 1] to semi-automatically determine the *PRNP *genotype. As positive controls, 8 samples of known genotypes representing all combinations of tested variants of each polymorphic locus and 1 NTC, should be included in every assay. Only if all of those samples are genotyped correctly, the genotypes of the unknown samples should be considered as reliable.

### Gel electrophoresis and elution

The 180-bp amplification product from sheep with known genotypes was visualized by 2% agarose multi-purpose molecular grade (Bioline, UK) gel electrophoresis, eluted in 20 μl with the Geneclean II kit (Bio101, USA), 1000 times diluted with 10 mM Tris-HCl pH 8 and used as a template during the optimalization of the genotyping assay. These purified PCR products were also used as positive controls in later assays. To avoid possible contamination of samples with these PCR controls, the setup of the control PCRs was performed after the setup of the sample PCRs, with the same PCR mix, while following standard procedures for good laboratory practise.

### PCR-RFLP

The PCR-RFLP assay is described by Peelman & Van Poucke [16], validated via a ring-test organized by the International Society of Animal Genetics in 2003–2004, and already used to genotype more than 3000 Belgian sheep [17].

### Direct sequencing

A *PRNP *amplicon of 315 bp, containing codons 136, 154 and 171, was amplified with PCR primers OariPRNPseqF 5'-GGAGGCTGGGGTCAAGGT-3' and OariPRNPseqR 5'-GGTGGTGGTGACTGTGTGTTG-3'. PCR was performed in the T3 Thermocycler (Biometra, Germany) in a total volume of 10 μl containing ~150 ng DNA, 500 nM of each primer, 0.8 mM dNTPs, 2 mM MgCl_2_, BioTaq buffer and 0.5 U BioTaq polymerase (Bioline, UK). The PCR program consists of a DNA denaturation step (5' at 95°C), followed by 30 amplification cycles (denaturation for 30" at 95°C, annealing for 30" at 63°C and elongation for 1' at 72°C), and a final elongation step for 10' at 72°C. Amplification products were visualized by 2% agarose multi-purpose molecular grade (Bioline, UK) gel electrophoresis and eluted in 20 μl with the Geneclean II kit (Bio101, USA). Approximately 200 ng of this purified PCR product was used in combination with 2 pmol OariPRNPseqR primer for a direct sequencing reaction with the Thermo Sequenase Cy5 Dye Terminator Sequencing Kit according to the manufacturers' instructions (Amersham Biosciences, Denmark). The reaction, consisting of 30 cycles (30" at 95°C, 30" at 58°C and 1'20" at 72°C), was performed in the T3 Thermocycler (Biometra, Germany) and analysed on the ALFexpress Sequencing system (Amersham Biosciences, Denmark).

## Results

### Dual fluorescent multiprobe assay optimalization

DNA was released from 8 sheep blood samples with known *PRNP *genotypes (VRQ/VRQ, VRQ/ARR, ARR/ARR, ARR/ARH, ARH/ARH, ARH/ARQ, AHQ/AHQ and ARR/AHQ), representing all combinations of tested variants of each polymorphic locus.

With those DNA samples as a template, the annealing temperature range of the primers was determined in which only the correct PCR fragment was amplified by a temperature gradient experiment in the iCycler iQ Real-Time PCR Detection System (data not shown). This experiment was performed with iQ Supermix, ~150 ng unpurified DNA and 200 nM primers in a total volume of 15 μl.

After probe quality testing, the hybridization temperature range was determined for each probe separately, in which highly specific hybridization occured without mismatch hybridization, within the optimal annealing temperature range of the primers. This was performed with a real-time temperature gradient experiment in the iCycler iQ Real-Time PCR Detection System (data not shown). The experiment was performed in a total volume of 15 μl with iQ Supermix, 200 nM primers and probe, and ~150 ng purified PCR product for 3 different samples (+/+, +/- and -/-) and H_2_O as NTC.

**Figure 1:**
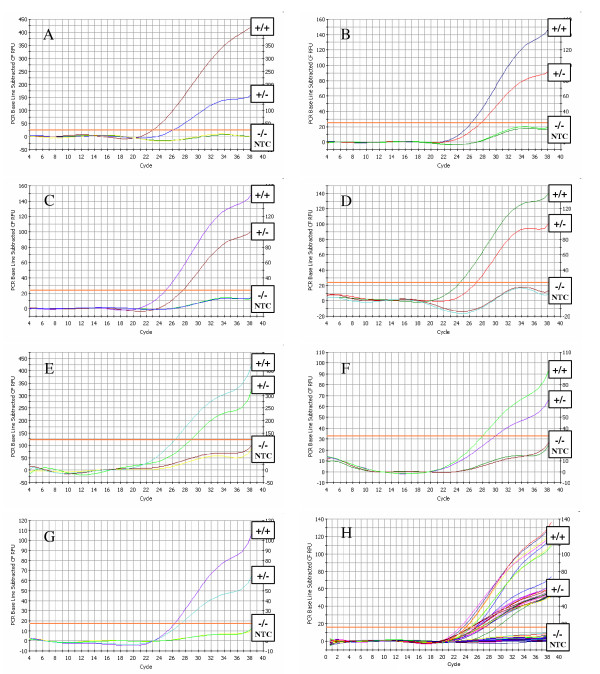
**(A-G) – Amplification plots obtained with the dual fluorescent multiprobe assay for sheep *PRNP *genotyping**. Amplification plots are shown for a homozygous positive (+/+), a heterozygote (+/-), a homozygous negative (-/-) and a no template control (NTC) with (A) A_136_-probe (FAM-labelled) in PCR 1, (B) V_136_-probe (HEX-labelled) in PCR 1, (C) R_154_-probe (TexasRed-labelled) in PCR 1, (D) H_154_-probe (Cy5-labelled) in PCR 1, (E) R_171_-probe (FAM-labelled) in PCR 2, (F) H_171_-probe (HEX-labelled) in PCR 2 and (G) Q_171_-probe (TexasRed-labelled) in PCR 2. (H) An amplification plot for 41 test samples and 9 control samples for the latter probe. For all amplification plots, unpurified DNA was used as a template.

Finally, considering the optimal hybridization temperature range of all probes, the optimal hybridization temperature and the primer and probe concentrations were determined for the 2 fluorescent multiprobe PCRs. For both closed tube PCRs an annealing/hybridization temperature of 62°C in combination with 400 nM primers and probes resulted in an accurate genotyping assay, even with unpurified DNA as a template. The obtained amplification curves from unpurified DNA samples with all possible outcomes for each probe are shown in Figure 1.

### Semi-automatic *PRNP *genotype determination

In order to speed up data analyses a Microsoft Excel-spreadsheet was developed for semi-automatic *PRNP *genotype determination [see Additional file 1]. Ct-values (in case of detection) or 'N/A' (in case of non-detection), obtained with the iCycler iQ Real-Time PCR Detection System Software version 3.0a (Bio-Rad Laboratories, USA), have to be entered (copy-paste) in the 'Ct-values Samples' tab of the spreadsheet for all probes of all test samples, together with their 'Run ID' and 'Sample ID', in order to generate the corresponding genotype. An impossible combination will result in a blank cell. The same thing should be done for the control samples in the 'Ct-values Controls' tab. The program automatically determines if the control samples are genotyped correctly. From the 'Results' tab, 'Sample ID' and genotype for every sample can be copy-pasted to other software programs, with a reminder if all control samples were genotyped correctly or not.

### Cross-validation study

**Figure 2:**
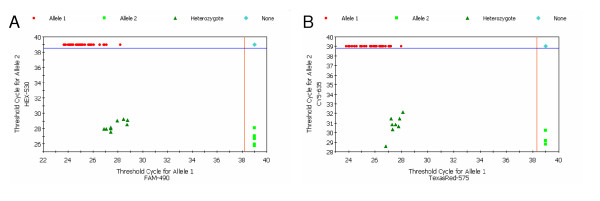
**(A-B) – Scatter plots based on Ct-values obtained with the dual fluorescent multiprobe assay for sheep *PRNP *genotyping**. In these scatter plots, Ct-values for 2 alleles of a specific codon are plotted on the xy axes from 50 sheep samples. (A) A_136_-probe (FAM-labelled) vs V_136_-probe (HEX-labelled) showing homozygous A_136_-genotypes (red closed dots), heterozygous genotypes (dark green closed triangles), homozygous V_136_-genotypes (light green closed squares), and a NTC (light blue closed diamond). (B) R_154_-probe (Texas-Red) vs H_154_-probe (Cy5-labelled) showing homozygous R_154_-genotypes (red closed dots), heterozygous genotypes (dark green closed triangles), homozygous H_154_-genotypes (light green closed squares), and a NTC (light blue closed diamond).

To evaluate the performance of the dual fluorescent multiprobe assay for *PRNP *genotyping in sheep, a total of 50 DNA samples that had been previously genotyped via PCR-RFLP [16,17], were tested in a blind manner. For every allelic variant homozygous and heterozygous genotypes were included at least 3 times. Concordant results were obtained for all 50 samples. Scatter plots for the allelic discrimination of both variants of codons 136 and 154, obtained via the Allelic Discrimination feature of the Data Analysis module in the Threshold Cycle Display Mode, are shown in Figure 2. Since the software is not able to distinguish more than 2 allelic variants, no plots are shown for codon 171. These results were also confirmed by direct sequencing. In addition, the assay was used to genotype more than 600 Belgian sheep within the TSE resistant breeding programme framework. No discrepancies with the rules of Mendelian inheritance were observed.

## Discussion

To minimize the risk ofTSE, breeding programmes of sheep to select resistant genotypes will be implemented in the near future in all EU countries [6]. An assay as described above will therefore be a vital instrument for a lot of molecular genetic laboratories to fulfill the need of genotyping hundreds or thousands of sheep. However, the most resistant ARR/ARR sheep are not 100% resistant to classical scrapie and BSE, and are furthermore susceptible to atypical scrapie [3,5,13]. Because scientists are searching for new resistance markers, it is likely that, based on new findings, future breeding programmes will be adapted. As a consequence, also the genotyping methods will have to be adapted, which can easily be done with this assay.

The assay can be performed on the iCycler iQ Real-Time PCR detection system (Bio-Rad) or on equally performing machines. It consists of 2 closed tube PCR reactions with the same PCR primers, spanning the codons 136, 154 and 171 of sheep *PRNP*. In PCR 1, polymorphisms at codons 136 (A/V) and 154 (R/H) are simultaneously detected in real time with 4 different dual-labelled fluorescent ASO probes. In PCR 2, polymorphisms at codon 171 (R/H/Q) are simultaneously detected in real time with 3 different dual-labelled fluorescent ASO probes. Although it was not the purpose of this assay, it is possible to distinguish homozygotes from heterozygotes, based on the shape of the amplification plots (Figure 1). These can not only serve as internal controls, but can also identify 'complex' *PRNP *genotypes [18].

A probe for the detection of the K_171 _variant was not included in the assay, because little is known about its association with TSE resistance/susceptibility [19]. If recommended, the probe can be included in PCR 2. The assay was not tested on sheep containing the K_171 _allele, but because of the low T_m _of the R_171_, H_171 _and Q_171 _probes with the K_171 _sequence, a K_171 _allele won't be detected with the current assay. In case of a homozygote no allele will be detected in PCR 2 and in case of a heterozygote only the other allele will be detected in PCR 2. However, the shape of the amplification plot will reveal that only 1 allele was detected. This will also be the case for every other mutation in the probe sequence, depending on the T_m _of the probes with the mutated sequence, which is inherent in a primer/probe based technique. Until now, no such problem was observed during routine genotyping of more than 600 Belgian sheep.

Although the sequencing method is still the golden standard for detecting all possible polymorphisms, it is a very time consuming and expensive technique not suitable for routine typing of large samples numbers in smaller service laboratories. The assay described here is an alternative method for routine typing of a defined number of polymorphisms. The capability of the iCycler to simultaneously excite and detect 4 different fluorophores, enables the use of 4 different probes in a single PCR. Only 2 PCR reactions, each with a total volume of 15 μl, are required for accurate genotyping calls for all 15 possible genotypes. Since the allelic variants are detected in real-time, no post-PCR manipulations are required, such as restriction digests and/or electrophoresis, reducing time, costs and possible carry-over contamination. Data can be copied to a Microsoft Excel-spreadsheet for semi-automatic determination of the genotype [see Additional file 1]. The control samples, which serve as external controls, are successfully analysed for many times during routine genotyping, proving the reproducibility of the assay.

Taking into account the 9 control samples, both PCR 1 and PCR 2 can be conducted in 1 single run using a well factor plate for calibration for less than 40 samples. For 40 samples or more (with a maximum of 87 samples per run), the assay should be performed in 2 runs (PCR 1 and PCR 2) using the experimental plate for calibration. Every run takes 1h25. When using robotic workstations and multiple real-time PCR detection systems this assay can easily be used in laboratories dealing with a large number of samples.

The assay is robust since it can be performed with unpurified DNA as template for PCR, as shown in Figure 1 and 2, although the amplification plots usually have lower Ct-values and higher RFU-values with purified PCR products (data not shown). Since the discrimination factor in the assay is whether or not a Ct-value is generated (the value itself is not important), the DNA concentration and quality don't influence the genotyping result, as long as a PCR product is generated. So, an extra DNA purification step could be included for bad preserved blood samples.

When applying the proposed primer/probe design strategy, the T_m _of the primers can be chosen randomly, as long as the T_m _of each probe with its perfect match target sequence is higher and its _av_T_m _with the other allelic variant (mismatch) is lower than the T_m _of the primers (see Table 1). A ΔT_m _of 2°C in both directions is sufficient to obtain the desired probe specificity for allelic discrimination (see Figure 1 and 2), without the need for expensive MGB- or LNA-probes [20,21]. We recommend to check every probe for fluorophore respons before starting any PCR optimization.

The performance of the assay was demonstrated via a cross-validation study. The correlation between the genotypes of 50 sheep generated with the dual fluorescent multiprobe assay and with PCR-RFLP and direct sequencing as reference methods was 100%. In addition, no discrepancies against the rules of Mendelian inheritance were observed during routine genotyping of more than 600 Belgian sheep.

## Conclusions

We have developed and validated a dual fluorescent multiprobe assay for robust, reliable and reproducible genotyping of few to many sheep samples in a fast, simple and cost-effective way, practicable in most standard equipped molecular genetic laboratories. The outlined primer/probe design strategy can also be applied for the detection of other polymorphisms or disease causing mutations.

## List of abbreviations

+/+     Homozygous positive

+/-     Heterozygous

-/-     Homozygous negative

A     Alanine

ARMS     Amplification Refractory Mutation System

ASO     Allele-Specific Oligonucleotide

_av_T_m     _Melting Temperature when annealed to Allelic Variant

BHQ     Black Hole Quencher

bp     basepairs

BSE     Bovine Spongiform Encephalopathy

CJD     Creutzfeldt-Jacob Disease

Ct     Threshold Cycle

DGGE     Denaturing Gradient Gel Electrophoresis

DNA     DeoxyriboNucleic Acid

EU     European Union

H     Histidine

LNA     Locked Nucleic Acid

MGB     Minor Groove Binding

N/A     Not Available

NTC     No Template Control

PCR     Polymerase Chain Reaction

*PRNP     *gene encoding PrP^C^

PrP^C     ^host encoded cellular prion protein

PrP^Sc     ^disease-causing isoform of PrP^C^

Q     Glutamine

R     Arginine

RFLP     Restriction Fragment Length Polymorphism

RFU     Relative Fluorescence Units

SNP     Single Nucleotide Polymorphism

T_m     _Melting Temperature

TSE     Transmissible Spongiform Encephalopathy

U     Unit(s)

V     Valine

vCJD     variant Creutzfeldt-Jacob Disease

vs     versus

## Competing interests

The author(s) declare that they have no competing interests.

## Authors' contributions

MVP designed the project and protocols involved, carried out the assays and drafted this manuscript. JV participated in primer/probe design and provided real-time PCR support. MM carried out the DNA preparations. AVZ and LJP participated in the design of the project.

## Pre-publication history

The pre-publication history for this paper can be accessed here:



## Supplementary Material

Additional File 1 A Microsoft Excel-spreadsheet for semi-automatic determination of the sheep PRNP genotype (based on codons 136, 154 and 171). Click here for file

## References

[B1] Prusiner SB (2004). Prion Biology and Diseases.

[B2] Prusiner SB (1998). The prion diseases. Brain Pathol.

[B3] Hunter N (2003). Scrapie and experimental BSE in sheep. Br Med Bull.

[B4] Bosque PJ (2002). Bovine spongiform encephalopathy, chronic wasting disease, scrapie, and the threat to humans from prion disease epizootics. Curr Neurol Neurosci Rep.

[B5] Houston F, Goldmann W, Chong A, Jeffrey M, Gonzalez L, Foster J, Parnham D, Hunter N (2003). Prion diseases: BSE in sheep bred for resistance to infection. Nature.

[B6] European Commission (2003). Decision 2003/100/EC of 13 February laying down minimum requirements for the establishment of breeding programmes for resistance to transmissible spongiform encephalopathies in sheep (Text with EEA relevance) (notified under document number C(2003) 498). Official Journal.

[B7] Junghans F, Teufel B, Buschmann A, Steng G, Groschup MH (1998). Genotyping of German sheep with respect to scrapie susceptibility. Vet Rec.

[B8] Yuzbasiyan-Gurkan V, Krehbiel JD, Cao Y, Venta PJ (1999). Development and usefulness of new polymerase chain reaction-based tests for detection of different alleles at codons 136 and 171 of the ovine prion protein gene. Am J Vet Res.

[B9] Belt PB, Muileman IH, Schreuder BE, Bos-de Ruijter J, Gielkens AL, Smits MA (1995). Identification of five allelic variants of the sheep PrP gene and their association with natural scrapie. J Gen Virol.

[B10] Zsolnai A, Anton I, Kuhn C, Fesus L (2003). Detection of single-nucleotide polymorphisms coding for three ovine prion protein variants by primer extension assay and capillary electrophoresis. Electrophoresis.

[B11] Buitkamp J, Semmer J (2004). A robust, low- to medium-throughput *PRNP *genotyping system. BMC Infect Dis.

[B12] Garcia-Crespo D, Oporto B, Gomez N, Nagore D, Benedicto L, Juste RA, Hurtado A (2004). PrP polymorphisms in Basque sheep breeds determined by PCR-restriction fragment length polymorphism and real-time PCR. Vet Rec.

[B13] Moum T, Olsaker I, Hopp P, Moldal T, Valheim M, Moum T, Benestad SL (2005). Polymorphisms at codons 141 and 154 in the ovine prion protein gene are associated with scrapie Nor98 cases. J Gen Virol.

[B14] Idaho Technology Inc. Downloads & Upgrades site. http://www.idahotech.com/downloads_up/index.html.

[B15] Ugozzoli LA, Chinn D, Hamby K (2002). Fluorescent multicolor multiplex homogeneous assay for the simultaneous analysis of the two most common hemochromatosis mutations. Anal Biochem.

[B16] Peelman LJ, Van Poucke M (2003). *PRNP *genotype frequency sampling of the most important sheep breeds in Belgium. Vl Diergeneeskd Tijdschr.

[B17] Roels S, Renard C, De Bosschere H, Geeroms R, Van Poucke M, Peelman L, Vanopdenbosch E (2004). Detection of polymorphisms in the prion protein gene in the Belgian sheep population: some preliminary data. Vet Q.

[B18] Dawson M, Warner R, Nolan A, McKeown B, Thomson J (2003). 'Complex' PrP genotypes identified by the National Scrapie Plan. Vet Rec.

[B19] Gombojav A, Ishiguro N, Horiuchi M, Serjmyadag D, Byambaa B, Shinagawa M (2003). Amino acid polymorphisms of PrP gene in Mongolian sheep. J Vet Med Sci.

[B20] Kutyavin IV, Afonina IA, Mills A, Gorn VV, Lukhtanov EA, Belousov ES, Singer MJ, Walburger DK, Lokhov SG, Gall AA, Dempcy R, Reed MW, Meyer RB, Hedgpeth J (2000). 3'-minor groove binder-DNA probes increase sequence specificity at PCR extension temperatures. Nucleic Acids Res.

[B21] Ugozzoli LA, Latorra D, Pucket R, Arar K, Hamby K (2004). Real-time genotyping with oligonucleotide probes containing locked nucleic acids. Anal Biochem.

